# Metabolic profiles associated with exposure to ambient particulate air pollution: findings from the Betula cohort

**DOI:** 10.3389/fpubh.2024.1401006

**Published:** 2024-08-13

**Authors:** Wasif Raza, Anders Öhman, Katja M. Kanninen, Pasi Jalava, Xiao-wen Zeng, Tosca O. E. de Crom, M. Arfan Ikram, Anna Oudin

**Affiliations:** ^1^Department of Public Health and Clinical Medicine, Umeå University, Umeå, Sweden; ^2^Department of Medical and Translational Biology, Umeå University, Umeå, Sweden; ^3^A.I. Virtanen Institute for Molecular Sciences, University of Eastern Finland, Kuopio, Finland; ^4^Department of Environmental and Biological Sciences, University of Eastern Finland, Kuopio, Finland; ^5^Department of Preventive Medicine, School of Public Health, Sun Yat-sen University, Guangzhou, China; ^6^Department of Laboratory Medicine, Lund University, Lund, Sweden

**Keywords:** air pollution, environmental epidemiology, metabolomics, dementia, cognitive disorders

## Abstract

**Introduction:**

Air pollution is a significant contributor to morbidity and mortality globally and has been linked to an increased risk of dementia. Previous studies within the Betula cohort in Northern Sweden have demonstrated associations between air pollution and dementia, as well as distinctive metabolomic profiles in dementia patients compared to controls. This study aimed to investigate whether air pollution is associated with quantitative changes in metabolite levels within this cohort, and whether future dementia status would modify this association.

**Methods:**

Both short-term and long-term exposure to air pollution were evaluated using high spatial resolution models and measured data. Air pollution from vehicle exhaust and woodsmoke were analyzed separately. Metabolomic profiling was conducted on 321 participants, including 58 serum samples from dementia patients and a control group matched for age, sex, and education level, using nuclear magnetic resonance spectroscopy.

**Results:**

No statistically significant associations were found between any metabolites and any measures of short-term or long-term exposure to air pollution. However, there were trends potentially suggesting associations between both long-term and short-term exposure to air pollution with lactate and glucose metabolites. Notably, these associations were observed despite the lack of correlation between long-term and short-term air pollution exposure in this cohort. There were also tendencies for associations between air pollution from woodsmoke to be more pronounced in participants that would later develop dementia, suggesting a potential effect depending on urban/rural factors.

**Discussion:**

While no significant associations were found, the trends observed in the data suggest potential links between air pollution exposure and changes in lactate and glucose metabolites. These findings provide some new insights into the link between air pollution and metabolic markers in a low-exposure setting. However, addressing existing limitations is crucial to improve the robustness and applicability of future research in this area. The pronounced associations in participants who later developed dementia may indicate an influence of urban/rural factors, warranting further investigation.

## 1 Introduction

Major Neurocognitive Disorder, often denoted dementia, is a significant public health challenge, with 10 million new cases reported annually, and its prevalence projected to triple between 2010 and 2050 according to the World Health Organization (1). Alzheimer’s disease (AD), an irreversible and progressive neurodegenerative disorder, alone accounts for a major part of all dementia cases and is the most prominent contributor to this global health issue. Given the substantial personal, social and economic burden associated with dementia care, and the lack of effective disease-modifying medications, it is crucial to identify and target potential modifiable risk factors as part of preventive policies (2).

Many well-established risk factors for dementia, such as hypertension, hyperlipidemia, and diabetes, are linked to lifestyle choices and can be modified to reduce the risk of disease. However, another modifiable risk factor that warrants attention at the population level is environmental: air pollution (3). Outdoor air pollution is composed of a complex mixture of toxic substances, including particulate matter (PM), irritant gases such as nitrogen dioxide (NO_2_), and heavy metals. PM, a mix of particles of various sizes and attributed from different sources, is considered responsible for many of the adverse health effects, both acute and chronic, associated with air pollution. Indeed, numerous studies have shown a positive association between daily mortality and ambient particle concentrations (4). Long-term exposure to ambient air pollutants, especially PM with a diameter of less than 2.5 micrograms (PM_2.5_), was estimated to have resulted in more than 4 million deaths worldwide in 2019 (5). While a substantial body of epidemiological research has established the association between air pollution exposure and cardiovascular diseases (6), the evidence regarding air pollution’s impact on cognitive decline, Alzheimer’s disease (AD), and other forms of dementia is more limited. Evidence is growing, however, and air pollution was suggested to be one of 12 established risk factors for dementia in the Lancet Commission Report Dementia prevention, intervention, and care in 2020 (3).

Despite indications that ambient air pollution may contribute to AD and other dementias, scientific evidence on this relationship remains limited, and the underlying biological mechanisms are not well understood. Given that the AD disease process begins many decades before the onset of symptoms during a lengthy preclinical period, it is of utmost importance to identify biomarkers of the disease to facilitate early diagnosis, prevention, and treatment (7). Metabolic profiling, which involves quantifying small molecules in cells, tissues, and biofluids, can be an effective tool for identifying new disease biomarkers and understanding disease mechanisms (8–10). Recent research on air pollution and non-targeted metabolomics suggests that the relationship between air pollution exposure and metabolic pathways primarily revolves around oxidative stress, inflammation, and steroid metabolism (11). A recent review provided a comprehensive overview of the utilization of untargeted metabolomics to detect alterations in metabolites or metabolic pathways linked to air pollution exposure and found 13 studies investigating short-term effects and 10 studies examining sub-chronic or long-term effects at the time of search in 2021 (12). Among the studies on long-term effects, there seemed to be considerable heterogeneity in exposure assessment methods. Additionally, local variations in major emission sources, such as traffic emissions and residential wood combustion have generally not been considered. We have previously observed associations between air pollution and dementia in the Betula cohort in Northern Sweden (13, 14). Some research in the same cohort has indicated altered concentrations of metabolites in individuals with dementia compared to healthy individuals (15, 16).

The main objective in the present study was to investigate whether exposure to air pollution was associated with quantitative changes of serum metabolite levels in the Betula cohort. A further aim was to explore whether these potential associations were dependent on future dementia status.

## 2 Materials and methods

### 2.1 Study population

The examination of the linear relationship among air pollution, metabolites, and covariates drew upon data from the Betula cohort, a prospective longitudinal study centered on aging, memory, and dementia established in 1988. Its primary aim was to detect early indicators of dementia by closely monitoring the health and cognitive progression of its participants over time. A total of 4,500 participants were randomly selected, with over-sampling of older adults, from approximately 125,000 residents in the Umeå municipality and assigned to six distinct cohorts at different time points. Since its inception, the Betula study has conducted six independent test waves (T1-T6), typically spanning about 1 week. These comprised comprehensive health examinations, cognitive assessments, and neurological evaluations. Additionally, biological samples were collected on two separate occasions within each test wave. An in-depth account of the recruitment process and the study’s design has been documented elsewhere (17). For the purposes of the present study, a subset of 321 participants were selected from test waves T4 (2003–2005) and T5 (2008–2010). These individuals were aged between 64 and 95 years at the time of the biological sample collection. The selection of the participants was based on available biofluid specimen of 1,144 individuals at T4. Among them, 136 individuals who were diagnosed with dementia case at T4 (2003–2005), T5 (2008–2010) and T6 (2013–2015) were selected. Additionally, 185 controls were matched with these dementia cases based on age, education, gender and vital status (alive or diseased) resulting in final study count of 321 individuals in present study.

### 2.2 Metabolomics profiling

A detailed description of the serum metabolic profiling procedure has been provided elsewhere (15). In brief, the metabolomic profiling involved the analysis of serum samples collected from both dementia patients and a control group matched for age, sex, and education level. This analysis was carried out using nuclear magnetic resonance (NMR) spectroscopy, a highly robust quantitative method, often referred to as NMR metabolomics or metabonomics (18). In total, 58 metabolites were quantified for each of the 321 samples using the Chenomx NMR Suite software package, version 8.01 (Chenomx Inc., Edmonton, Canada).

### 2.3 Air pollution exposure assessment

#### 2.3.1 Long-term exposure

To estimate exposure to PM_2.5_ and Black Carbon (BC), we relied on data for the years 1990, 2000, and 2010, which were computed by the Swedish Meteorological and Hydrological Institute (SMHI) using a comprehensive methodology specified elsewhere (19). In short, this involved the utilization of a wind model and a Gaussian air quality dispersion model with detailed input data. Notably, the principal local contributors to PM_2.5_ emissions encompassed road traffic and residential wood burning.

Concerning PM_2.5_ and BC from road traffic, they were modelled separately for exhaust and wear-and-tear. Road networks were meticulously described with a high degree of detail, and the recorded traffic flow data for both heavy and light vehicles were collected separately by SMHI for major roadways, with additional data being modelled for less travelled routes. Additionally, the composition of the vehicle fleet was derived from the National Vehicle Registry and categorized into passenger cars (petrol, diesel, ethanol, gas), light commercial vehicles (petrol and diesel), heavy goods vehicles (petrol and diesel), and buses (diesel, biogas, and ethanol). Emission factors for exhaust emissions from various vehicle types, taking into account different speeds and driving conditions, were calculated based on the Handbook Emission Factors for Road Transport (HBEFA) 3.1 (20).

For the assessment of PM_2.5_ from residential heating, SMHI utilized a comprehensive emission inventory based on information gathered from chimney sweepers, collected by the Department for Occupational and Environmental Medicine at Umeå University. This inventory allowed emissions to be represented as point sources, encompassing a total of 10,287 appliances from the period 2006 to 2009 within the assessment area. The inventory underwent validation through a monitoring campaign conducted in and around Umeå, with a particular focus on areas significantly impacted by small-scale residential heating. Additionally, a survey was carried out to gather information on wood consumption and firing habits, enabling the estimation of average wood consumption for various heating appliances. Omstedt et al. (21) provide further insights into emission factors, inventory validation, and survey details. Within our study area, residential wood burning was the predominant source of local particle emissions from residential heating, and as such, this particle source is henceforth referred to as “residential wood burning.”

The original spatial resolution of the model grids was 3,200 m x 3,200 m but was progressively refined to 50 m x 50 m in areas with increased urbanization. To estimate historical air pollution levels, we performed a backward extrapolation of the modelled PM_2.5_ and BC concentrations to the levels observed at T3. This was achieved by applying scaling factors for each year, using 2009 as the baseline year. For the years spanning 1998 to 2000, the annual mean concentrations of PM_2.5_ and BC were modelled to be 6.75 μg/m^3^ and 0.49 μg/m^3^, respectively.

The pollutant measures included in the analysis were: PM_2.5_ related to traffic emissions (PM_2.5_exhaust), residential wood burning (PM_2.5_ wood), road wear and tear PM_2.5_ non-exhaust, all local sources of PM_2.5_ together with regional background concentrations (PM_2.5_ total), BC-concentrations associated with traffic (BC traffic), residential wood burning (BC wood), and all local sources of BC combined (BC total). These measures served as indicators of long-term air pollution exposure.

#### 2.3.2 Short-term exposure

We also used data on 24 hourly mean concentrations of PM_10_ with a diameter of less than 10 micrograms (PM_10_) from the 1st of January 2003 to the 31st of December 2010 as a measure of short-term air pollution exposure. Data from a measurement station in the study area describing urban background concentrations was downloaded from SMHI’s server.^1^

### 2.4 Statistical analysis

All data are presented in the form of participant characteristics, or covariates, based on mean concentrations and standard deviations of PM_2.5_ exhaust and PM_2.5_ wood.

To explore the relationship between long-term exposure to air pollutants and serum metabolites, we employed Spearman’s correlation analysis. All analysis was then stratified by future dementia status (yes/no). This variable describes whether a participant receives a dementia diagnosis at any time after the study period extending from memory testing to the end of follow up period in 2014. The relationship between long-term exposure to PM_2.5_ and serum metabolites was, furthermore, analyzed using linear regression analysis and adjusted for age at sampling date, and sex.

The relationship between measurements of daily averages of PM_10_ at the sampling date and serum metabolites, using biological samples from 34 participants (participants with missing PM_10_ measurements were excluded), was also analyzed with linear regression. Analyses were adjusted for participants’ age and sex.

All analyses were conducted using R version 3.4.8.

The study was approved by the ethics review authority with Dnr: 2022–04608-01 and written informed consent was given by all Betula participants. The researchers analyzing the data did not have access to information that could identify individual participants.

## 3 Results

**Figure 1:**
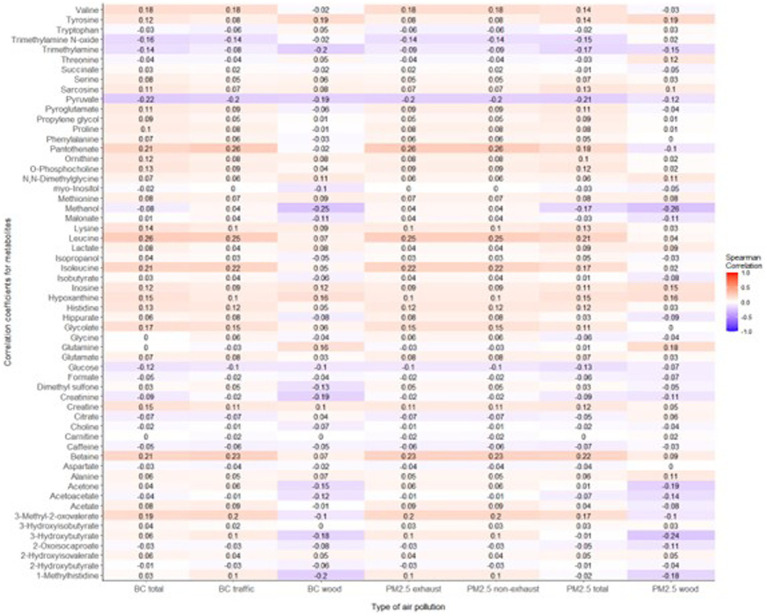
Tile plot displaying correlations between the investigated air pollutants (long-term exposure) and serum metabolites. A mosaic of light blue shades indicates a weak negative correlation between pollutants and metabolites while the light shades of red tones emphasizes the weak positive correlation between pollutants and metabolites.

**Figure 2:**
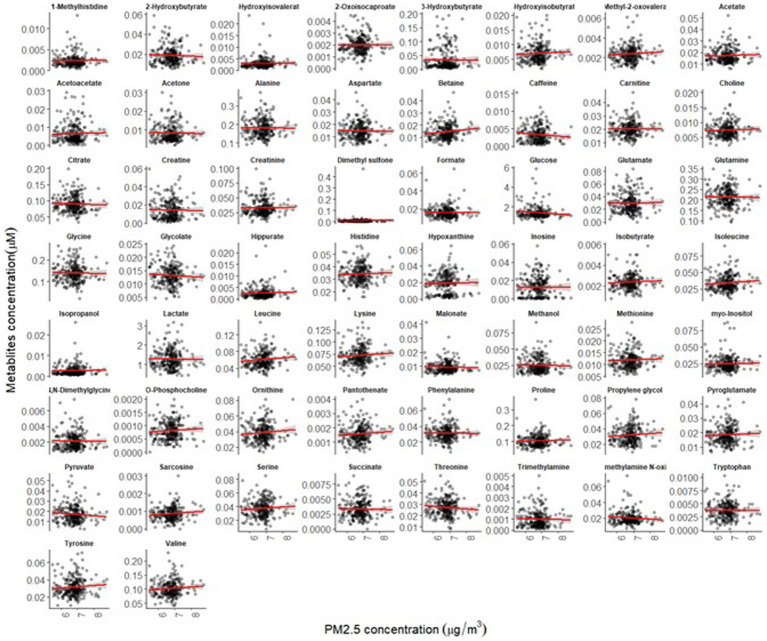
Simple linear regression analysis showing the association between long-term exposure to PM_2.5_-total and the 58 investigated serum metabolites (the red lines illustrate the estimated slopes).

**Figure 3:**
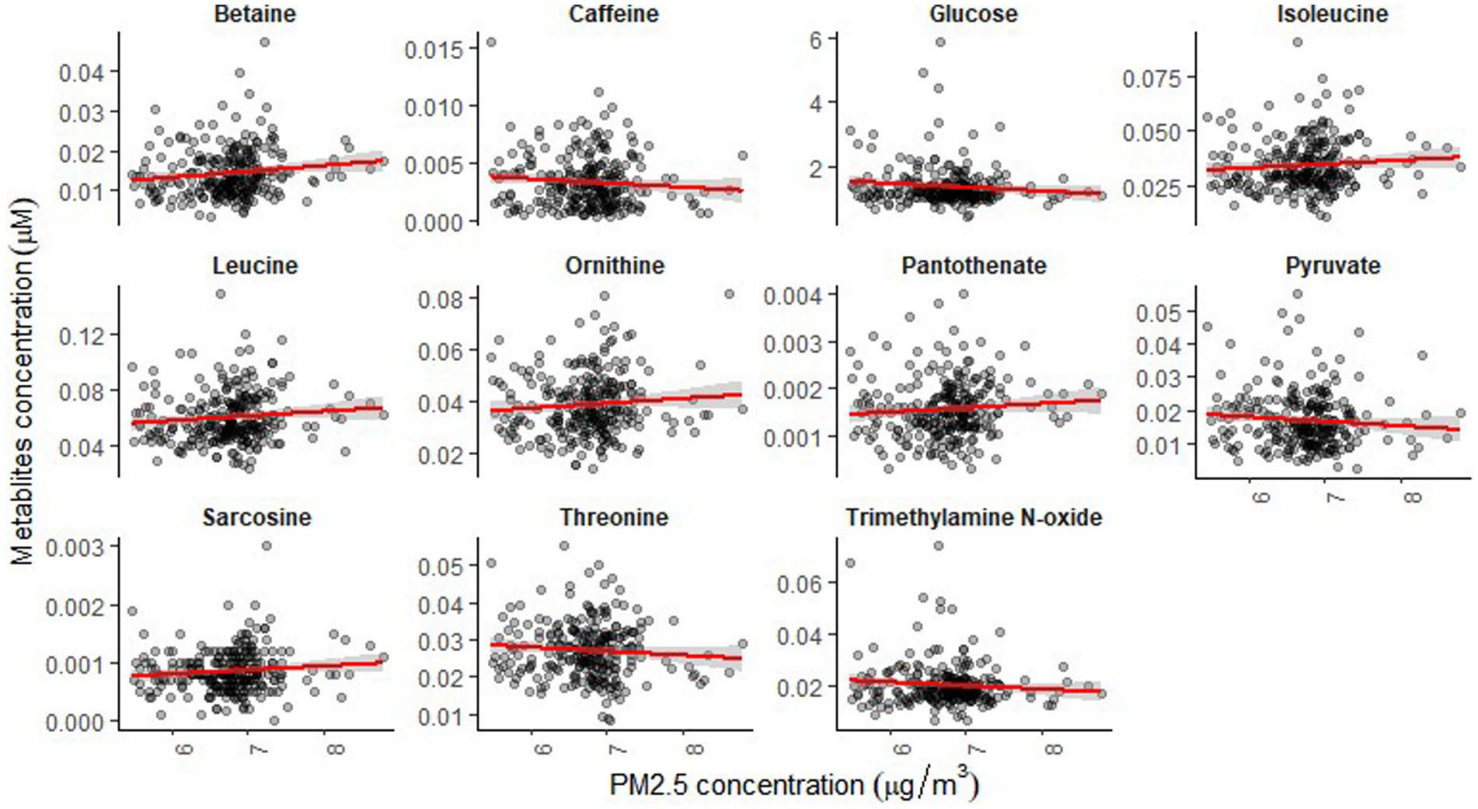
Simple linear regression analysis showing the association between long-term exposure to PM_2.5_-total and the serum metabolites for *p*-value <0.2.

**Figure 4:**
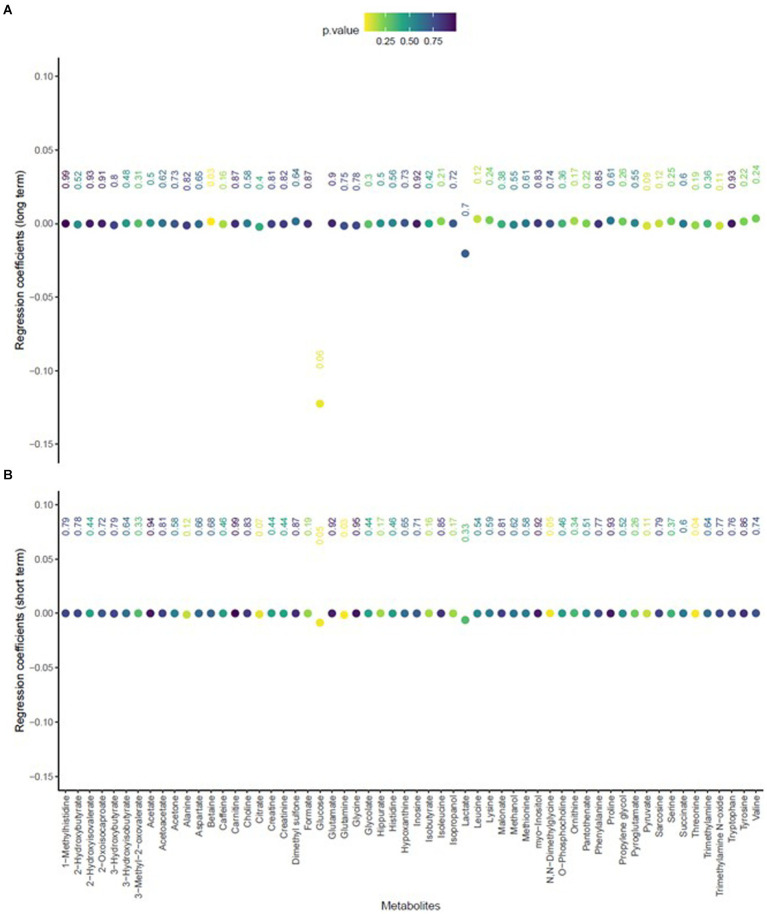
Point plot presenting the regression coefficients (y-axis) of the association of total PM_2.5_ exposure, including long-term **(A)** and short-term **(B)**, with each of the investigated serum metabolites (x-axis), adjusted for age and sex.

The mean age of participants at recruitment was 79.9 years. Within the cohort of 321 participants, our study sample consisted of 136 individuals with dementia and 185 healthy individuals. Descriptive data for all variables, including the mean concentrations of PM_2.5_ exhaust and PM_2.5_ wood, are presented in Table 1. A tile plot illustrating the correlations between pollutants and serum metabolites is presented in Figure 1. In general, we observed very weak correlations between pollutants and metabolites. For instance, there were weak positive correlations between leucine and PM_2.5_ total (rs = 0.21, *p*-value = 0.02), PM_2.5_ exhaust (rs = 0.26, *p*-value = 0.01), PM_2.5_ non-exhaust (rs = 0.256, *p*-value = 0.01), BC total (rs = 0.26, *p*-value = 0.00), and BC traffic (rs = 0.25, *p*-value = 0.01). Conversely, a weak negative correlation was observed between pyruvate and PM_2.5_ total (rs = −0.21, *p*-value = 0.03), PM_2.5_ exhaust (rs = −0.2, *p*-value = 0.03), PM_2.5_ non exhaust (rs = −0.2, *p*-value = 0.03), BC total (rs = −0.22, *p*-value = 0.02), and BC traffic (rs = −0.2, *p*-value = 0.03). Results from the simple linear regression analysis (Figure 2) were in line with these findings, the estimated slopes were imprecise. In Figure 3, the metabolites from Figure 2 for which the *p*-value was less than 0.2 were selected for closer presentation. Figure 4 is a point plot presenting the regression coefficients for long-term PM_2.5_ exposure (A) and short-term PM_2.5_ exposure (B), with each of the investigated serum metabolites adjusted for age and sex. Most estimates were very close to zero, with the exception of Glucose (*β* = −1.22, *p*-value = 0.06 and) and, to some extent Lactate (*β* = −0.02, p-value = 0.7), which seemed to be negatively correlated with total PM_2.5_ especially for long-term exposure but also a tendency of signal for the same metabolites with short-term exposure, at least visually (Figures 4A,B). The corresponding estimates for short term exposure of PM_10_ for glucose and lactose were (*β* = −0.009, *p*-value = 0.05) and (*β* = −0.006, *p*-value = 0.33) respectively.

**Table 1:** Distribution of Population characteristics at recruitment according to mean PM_2.5_ exposure to vehicle exhaust and residential wood burning [number of individuals (N) and dementia cases (n), Mean (standard deviation, SD)].

			PM_2.5_ (μg/m^3^)	
			Wood burning	Vehicle exhaust
		N (n)	Mean (SD)	Mean (SD)
Age	64–74	84 (33)	0.88 (0.22)	0.15 (0.14)
	75–85	191 (83)	0.84 (0.24)	0.19 (0.19)
	> 85	46 (20)	0.89 (0.27)	0.203 (0.24)
Sex	Female	204 (87)	0.86 (0.24)	0.18 (0.18)
	Male	117 (49)	0.86 (0.24)	0.19 (0.20)
Education	Compulsory	209 (108)	0.84 (0.24)	0.18 (0.19)
	High School	35 (14)	0.91 (0.21)	0.17 (0.11)
	University	28 (12)	0.99 (0.24)	0.21 (0.24)
Smoking	No	207 (81)	0.85 (0.26)	0.18 (0.18)
	Yes	105 (47)	0.87(0.20)	0.20 (0.20)
BMI^*^	Overweight	165 (61)	0.84 (0.24)	0.19 (0.20)
	Normal or Underweight	140 (69)	0.88 (0.25)	0.18 (0.16)
Medication				
Anti-hypertensive	No	169 (71)	0.86 (0.25)	0.19 (0.19)
	Yes	130 (51)	0.86 (0.22)	0.18 (0.18)
Anti-Alzheimer’s	No	305 (120)	0.86 (0.24)	0.18 (0.18)
	Yes	16 (16)	0.87 (0.20)	0.24 (0.28)
Lipid lowering agents	No	271 (112)	0.86 (0.24)	0.185 (0.19)
	Yes	50 (24)	0.84 (0.21)	0.18 (0.17)
Anti-diabetics	No	295 (127)	0.86 (0.24)	0.19 (0.19)
	Yes	26 (9)	0.86 (0.28)	0.14 (0.12)
Anti-thrombocytes	No	185 (77)	0.86 (0.26)	0.18 (0.16)
	Yes	136 (59)	0.86 (0.21)	0.195 (0.21)
Diuretics	No	221 (96)	0.87 (0.23)	0.180 (0.18)
	Yes	100 (40)	0.85 (0.25)	0.190 (0.20)
Vitamin intake^†^	No	237 (96)	0.87 (0.25)	0.177 (0.18)
	Yes	84 (40)	0.84 (0.21)	0.203 (0.20)

^*^Overweight: >25 kg/m^2^. ^†^Normal or underweight: ≤25 kg/m^2^.

**Figure 5:**
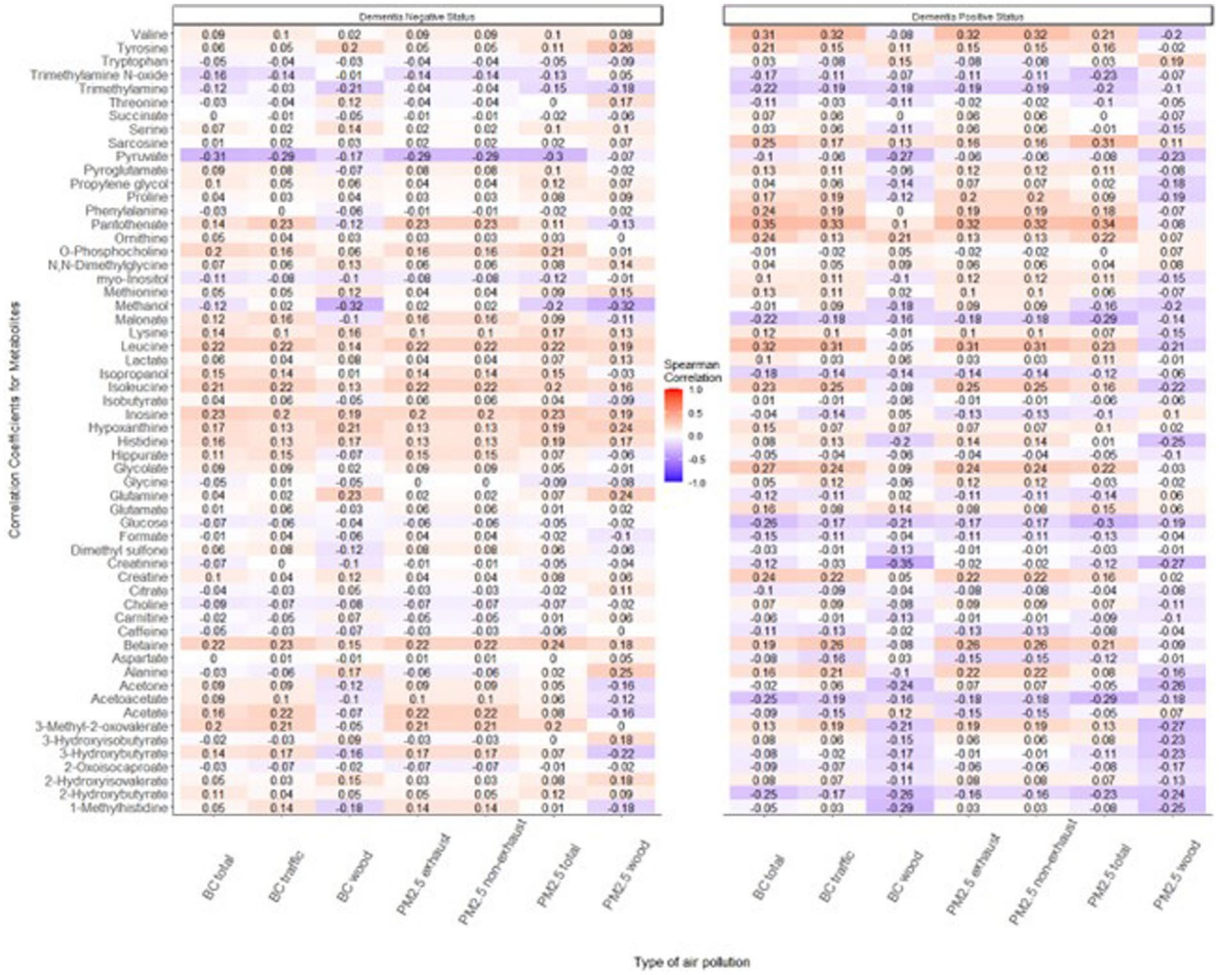
Tile plot displaying correlations between pollutant types and metabolites, stratified by future dementia status. A mosaic of light blue shades indicates a weak negative correlation between pollutants and metabolites while the light shades of red tones emphasize the weak positive correlation between pollutants and metabolites among those classified for their future dementia status.

When stratifying the analysis by future dementia status, the correlations were still weak (Figure 5). Although inconsistent (*p*-values were > 0.05), there was a tendency for the patterns to differ between the two groups, however. Among study participants who later developed dementia, the associations were to a larger extent negative for PM_2.5_ wood than among control participants who did not receive a dementia diagnosis during follow-up.

## 4 Discussion

### 4.1 Main findings

In this study, we did not find strong evidence supporting association between exposure to ambient particulate air pollution and serum metabolites, in a low exposure setting in Northern Sweden. The lack of clear associations was seen for both long-term and short-term exposure to air pollution. There were tendencies for the Glucose and Lactate metabolites to be negatively associated with long-term-, and possibly also short-term exposure to air pollution, however. While these observations could potentially be a result of random chance due to multiple comparisons, they are still considered to be noteworthy.

### 4.2 Identifying metabolites

Findings from previous studies are somewhat mixed, and there seems to be substantial variation regarding which metabolites have been found to be associated with air pollution exposure. A study conducted in the USA employed untargeted metabolomics, which has the capability to identify metabolomic signals associated with traffic exposure and utilized weekly mean pollutant concentrations (22). Notably, arginine and histidine were among the 10 validated metabolites found to be associated with traffic exposure. In a separate study conducted in the Netherlands, a narrower exposure window of 5 h was employed, and metabolite profiling was conducted using 493 blood samples collected from 31 volunteers (23). Tyrosine, guanosine, and hypoxanthine were among the metabolites associated with air pollution exposure. In another study from the USA, metabolic perturbations associated with daily mean exposures to traffic-related air pollutants among 180 participants (24). Here, histamine and uracil were found to be associated with carbon monoxide, nitrogen dioxide, and elemental carbon. In a Chinese study, the two primary metabolic signatures were detected: comprised lipids and dipeptides, polyunsaturated fatty acids, taurine, and xanthine (25). Metabolites in both groups exhibited a decline during the 2008 Beijing Olympics when air pollution concentrations decreased, followed by an increase after the Olympics when air pollution returned to its usual (high) levels.

In a recent review, 47 studies was found where untargeted metabolomics was used to explore the impact of air pollution on the human metabolome (11). Thirty-five metabolites, including hypoxanthine, histidine, serine, aspartate, and glutamate, consistently showed associations with multiple pollutants in at least 5 independent studies.

The present study observed tendencies for the metabolites Glucose and Lactate to be associated with long-term, and possibly, short-term PM-concentrations when adjusted for age and sex. It should be noted that there were many comparisons in the study, and a certain number of associations hence should be expected to be observed due to chance. However, the fact that the tendency of the associations is observed for both long- and short-term exposure is intriguing, since there is no reason for the long-term and short-term exposure measures to correlate in the present study.

### 4.3 Possible pathways

Commonly affected pathways identified the recent review of 47 studies involved oxidative stress and inflammation, such as glycerophospholipid metabolism, pyrimidine metabolism, methionine and cysteine metabolism, tyrosine metabolism, and tryptophan metabolism (11). In the present study, a limited number of metabolites were analyzed, and only two metabolites seemed to be associated with air pollution exposure. Pathway analyses were thus not applicable.

Original studies also support the association between both oxidative stress and inflammation pathways and traffic and have also highlighted an association between traffic exposure and leukotriene and vitamin E metabolism (22). Significant alterations in the blood metabolome correlating with fluctuations in air pollution levels, some of which were linked to acute health effects, have also been identified (23). Pathway analysis conducted in the same original study demonstrated augmentation in the course of eight pathways, with a notable emphasis on tyrosine metabolism (24). Additionally, in a U.S. study, DNA damage and repair, nutrient metabolism, and acute inflammation were all linked to traffic-related air pollutants and pyrimidine metabolism, as well as the carnitine shuttle which assists the active transportation of long chain fatty acid from the blood stream to the mitochondria for energy production (24). However, authors of the review article concluded that future efforts should prioritize validation through hypothesis-driven protocols and advancements in metabolic annotation and quantification techniques (11).

Regarding the present study’s results, Glucose and Lactate’s tendency toward an association with both long-term and short-term to ambient PM is also interesting for the discussion of metabolic pathways. For instance, evidence on the association between ambient air pollution and diabetes, for which Glucose is a key factor, is increasing, and air pollution appears to be associated with dysregulation of glucose metabolism (26). Additionally, Lactate, a crucial metabolic substrate, exerts its regulatory impacts on immune response depending on the cell type and pathway by which lactate is produced or metabolized, which can either hinder or enhance the inflammatory response (27). This metabolite also functions as an intercellular and inter-tissue redox signaling molecule and plays a key role in supplying energy for oxidative metabolism across various tissues, contributing to the preservation of redox homeostasis and the overall integrity of both tissues and the entire organism (28). Given that oxidative potential in particulate air pollution is often stated as one of the key mechanistic pathways for various diseases, the tendency of an association with Lactate should be further investigated in future studies. The findings related to Lactate are also interesting given emerging evidence indicating that particulate air pollution may affect the microbiota (29).

### 4.4 Dementia diagnosis

In the present study, the correlation between various PM air pollutants, especially those related to wood combustion, and metabolites appeared more pronounced among individuals who subsequently developed dementia. These findings are not conclusive however, with high *p*-values, but could be seen as hypothesis generating. To our knowledge, differential associations due to future dementia diagnosis have not been investigated, or seen, before. The finding may imply a differential impact of exposure on susceptible groups, prompting inquiries into the varying metabolomic response among those who later experience dementia. Moreover, this finding aligns, to some extent, with prior results from the Betula cohort, which have indicated clearer associations between air pollution and dementia among more susceptible groups (30), and associations between exposure to woodsmoke and dementia (14). We have also earlier seen in the Betula cohort that the connection between particulate air pollution and dementia exhibited greater strength among participants with the APOE ε4 allele and among those with lower scores on odor identification ability (30).

We primarily focused on metabolic markers from the serum metabolome and their associations with air pollution exposure. The metabolites in our study were previously analyzed using nuclear magnetic resonance spectroscopy both in serum and saliva, revealing statistically significant models that distinguished dementia patients from controls (15, 16). Dementia patients showed elevated levels of acetic acid, histamine, and propionate, with decreased levels of dimethyl sulfone and succinate. These metabolites, along with others, have relevance in AD pathways, dietary influences, and periodontal status. While providing valuable insights, our study did not explore a broader range of metabolites. Future research could expand this scope and consider stratifying analysis by other diseases. Additionally, threonine emerged as particularly significant in both dementia and pre-dementia groups, impacting multiple metabolic pathways. However, only two metabolites (lactate and glucose) stood out in the present study, limiting pathway analysis. Future research could include more metabolites and consider other disease stratifications.

### 4.5 Methodological considerations

The study has strengths and limitations to consider. A major strength of our study is that we were able to uniquely investigate traffic exhaust, non-exhaust emissions, and wood smoke separately, which are the major sources of locally emitted air pollution in the area. We furthermore achieved a spatial resolution as fine as 50×50 meters in urban areas, allowing for precise capture of local emissions. Additionally, the study utilizes both long-term and short-term exposure estimates, providing a robust analysis of the impact of air pollution.

This study is furthermore subject to certain limitations that should be considered when interpreting the findings. A significant limitation relates to exposure misclassification, a common challenge in studies on air pollution and health, as people are not always at their homes or may spend minimal time outdoors, which is where air pollution is modelled. While we employed advanced models to estimate long-term exposure to specific sources of particulate air pollution, individual-level variations in factors such as residential mobility and wood firing habits, which can influence personal and neighborhood-level exposure, were not accounted for. Regarding short-term exposure, only one measurement station was used, which is also an evident source of misclassification although being standard approach. While this could potentially introduce bias into our results, the applied approach to exposure assessment is considered state of the art in the field of air pollution epidemiology, and similar approaches are used in other studies (31, 32).

Another potential limitation pertains to the lack of knowledge surrounding relevant exposure windows. We used the annual mean as a marker for long-term exposure to air pollution and the daily mean as a marker for short-term exposure to air pollution. Metabolites are often influenced quite directly, and short-term exposure may, therefore, carry more significance than long-term exposure in this context. Generally, the annual mean serves as a reasonably valid proxy for exposure over the past several months. Daily mean concentrations can vary substantially from day to day, however. The choice of time windows may, thus, affect the results.

Residual confounding is another aspect to consider in observational studies. In the present study, we had access to data on several potential confounding factors, such as education level and smoking status. However, we refrained from adjusting for such potential confounding factors because the correlations between the pollutants and the metabolites were markedly low and there was limited statistical power to include potential confounding factors into the models.

Finally, the results generated from this study may not be universally applicable, as they are based on a specific population in Northern Sweden, in an area with generally quite low levels of air pollution concentrations. Factors like socio-economic status, lifestyle, demographics, and local environmental conditions can vary significantly between regions and populations. With this, caution should be exercised when attempting to generalize these findings to different geographic areas and demographic groups. Air pollution itself is an intricate mix subject to temporal and spatial variation, posing an additional challenge for assessing.

## 5 Conclusion

We found tendencies for the Glucose and Lactate metabolites to be associated with long-term, and possibly also short-term, exposure to particulate air pollution. Additionally, we observed tendencies for associations between PM_2.5_ from residential wood burning and the investigated serum metabolites mainly in participants who later developed dementia. In future research, it is essential to address existing limitations to enhance robustness and applicability to achieve more conclusive results, with sufficient statistical power. Continuous efforts to combat air pollution and implement long-term strategies are imperative for the well-being of both local and global populations. With the ultimate goal furthermore of establishing strategies for the early identification of individuals at risk of AD and to identify novel targets for preventive measures, these findings, although not conclusive, can possibly generate hypotheses for future studies. The findings may furthermore contribute to the growing body of evidence illustrating the potential for enhanced health due to improved air quality.

## Data availability statement

The datasets presented in this article are not readily available because they contain personal sensitive information and thus cannot be shared openly. Requests to access the datasets should be directed to the Betula steering group: https://www.umu.se/forskning/projekt/betula---aldrande-minne-och-demens/.

## Ethics statement

The studies involving humans were approved by Ethics review authority in Sweden with Dnr: 2022-04608. The studies were conducted in accordance with the local legislation and institutional requirements. The participants provided their written informed consent to participate in this study.

## Author contributions

WR: Formal analysis, Writing – original draft. AÖ: Writing – review & editing, Writing – original draft. KK: Conceptualization, Funding acquisition, Writing – review & editing. PJ: Funding acquisition, Writing – review & editing. X-wZ: Writing – review & editing. TC: Writing – review & editing. MI: Writing – review & editing. AO: Conceptualization, Data curation, Funding acquisition, Methodology, Project administration, Supervision, Writing – original draft, Writing – review & editing.

## Footnotes

^1^ http://www.smhi.se/datavardluft
